# Occurrence and Persistency of Conduction Disturbances during Transcatheter Aortic Valve Implantation

**DOI:** 10.3390/medicina57070695

**Published:** 2021-07-07

**Authors:** Thomas T. Poels, Elien B. Engels, Suzanne Kats, Leo Veenstra, Vincent van Ommen, Kevin Vernooy, Jos G. Maessen, Frits W. Prinzen

**Affiliations:** 1 Department of Cardiothoracic Surgery, Maastricht University Medical Center, P.O. Box 5800 Maastricht, The Netherlands; suzanne.kats@mumc.nl (S.K.); j.g.maessen@mumc.nl (J.G.M.); 2 Department of Physiology, Faculty of Health, Medicine and Life Sciences, CARIM School for Cardiovascular Diseases, P.O. Box 616 Maastricht, The Netherlands; elienengels@gmail.com (E.B.E.); frits.prinzen@maastrichtuniversity.nl (F.W.P.); 3 Department of Cardiology, Maastricht University Medical Center, P.O. Box 5800 Maastricht, The Netherlands; l.veenstra@mumc.nl (L.V.); v.van.ommen@mumc.nl (V.v.O.); kevin.vernooy@mumc.nl (K.V.)

**Keywords:** transcatheter aortic valve implantation, left bundle branch block, electrocardiogram

## Abstract

*Background and Objectives*: Conduction disturbances such as left bundle branch block (LBBB) and complete atrio-ventricular block (cAVB) are relatively frequent complications following trans-catheter aortic valve implantation (TAVI). We investigated the dynamics of these conduction blocks to further understand luxating factors and predictors for their persistency. *Materials and Methods*: We prospectively included 157 consecutive patients who underwent a TAVI procedure. Electrocardiograms (ECGs) were obtained at specific time points during the TAVI procedure and at follow-up until at least six months post-procedure. *Results:* Of the 106 patients with a narrow QRS complex (nQRS) before TAVI, ~70% developed LBBB; 28 (26.4%) being classified as super-transient (ST-LBBB), 20 (18.9%) as transient (T-LBBB) and 24 (22.6%) as persistent (P-LBBB). Risk of LBBB was higher for self-expandable (SE) than for balloon-expandable (BE) prostheses and increased with larger implant depth. During the TAVI procedure conduction disturbances showed a dynamic behavior, as illustrated by alternating kinds of blocks in 18 cases. Most LBBBs developed during balloon aortic valvuloplasty (BAV) and at positioning and deployment of the TAVI prosthesis. The incidence of LBBB was not significantly different between patients who did and did not undergo BAV prior to TAVI implantation (65.3% and 74.2%, respectively (*p* = 0.494)). Progression to cAVB was most frequent for patients with preexisting conduction abnormalities (5/34) patients) and in patients showing ST-LBBB (6/28). *Conclusions*: During the TAVI procedure, conduction disturbances showed a dynamic behavior with alternating types of block in 18 cases. After a dynamic period of often alternating types of block, most BBBs are reversible while one third persist. Patients with ST-LBBB are most prone to progressing into cAVB. The observation that the incidence of developing LBBB after TAVI is similar with and without BAV suggests that a subgroup of patients has a substrate to develop LBBB regardless of the procedure.

## 1. Introduction

Transcatheter aortic valve implantation (TAVI) is a treatment option for patients with severe aortic valve stenosis who do not qualify for surgical aortic valve replacement (AVR) [1,2]. Despite its proven clinical benefits, conduction disturbances such as left bundle branch block (LBBB) remain one of the observed complications following TAVI. Previous studies reported that LBBB occurs in 7–65% of the cases and that it persists in about one third of these cases [3,4,5,6,7,8]. Several studies showed that LBBB is associated with a higher mortality rate, presumably related to dyssynchrony-induced heart failure and development of atrioventricular (AV) block [5,9,10]. Luxating factors for the occurrence of LBBB, such as implantation depth, low ratio of annulus/balloon or annulus/prosthesis and the type of prosthesis, have been reported in post-procedural analyses [11]. Oedema and permanent compression by the prosthesis with resulting damage and/or ischemia [8,11,12] have been suggested as potential causes for conduction disturbances following TAVI. Little prospective research has been performed for better understanding of the peri-operative luxating factors of conduction disturbances and predictors for persistency. In a small prospective research using Medtronic Corevalve prostheses, Nuis et al. showed that more than half of the LBBBs occur before prosthesis implantation, but long-term persistency was not investigated [8].

We sought to investigate the incidence, timing and dynamics of conduction disturbances in patients undergoing TAVI with emphasis on the development of LBBB to better understand the role of luxating factors.

## 2. Materials and Methods

### 2.1. Study Population

All consecutive patients who underwent a TAVI between April 2014 and June 2016 in the Maastricht University Medical Center were included in this study. Exclusion criteria were a pre-existing permanent pacemaker implantation (PPI) and implantation of a prior aortic valve prosthesis.

Our objective was to study at least 50 patients with a narrow QRS complex (nQRS) at baseline in order to analyze trends of conduction disturbances during TAVI. Taking into account patients with a wide QRS at baseline and to compensate for missing data and lost to follow-up, a total number of 150 patients was considered between April 2014 and June 2016. Patients were included from April 2014 to June 2016. Electrocardiographic data were prospectively collected in a central registry.

### 2.2. Study Design

Electrocardiograms (ECGs) were obtained preoperatively, postoperatively, before discharge and at follow-up after 6–12 months following TAVI.

Continuous 12-lead ECG (cECG) was recorded during key stages of the TAVI procedure as follows:

1. at baseline, within one minute prior to start of the procedure,
2. after advancing the soft guidewire through the aortic valve,
3. after placing the stiff wire through the aortic valve,
4. after positioning the valvuloplasty balloon through the aortic valve,
5. after performing the balloon aortic valvuloplasty (BAV),
6. after positioning the TAVI prosthesis in the native valve,
7. after deployment of the TAVI prosthesis and
8. at the end of the procedure after removal of all catheters.

All additional BAVs, both positioning and dilatation, following TAVI deployment, were also registered.

All ECGs were reviewed for ventricular conduction disturbances such as LBBB, right bundle branch block (RBBB), intraventricular conduction disturbance (IVCD) and complete atrioventricular block (cAVB). According to the established Strauss criteria, LBBB was defined as a QRS duration of >140 ms (men) or >130 ms (women), QS or rS in leads V1 and V2 and mid-QRS notching or slurring in 2 of leads V1, V2, V5, V6, I, and aVL [13]. RBBB was defined as a QRS complex ≥120 ms with a triphasic QRS complex in V1 together with a dominant S wave in leads I and V6 [14]. IVCD was defined as any other broad QRS complex ≥120 not defined as a LBBB or RBBB.

Any new bundle branch block (BBB) on the cECG which had disappeared on the first postoperative ECG was defined as super-transient (ST). If a new BBB or cAVB was present postoperatively but disappeared at discharge or 6–12 months follow-up it was defined as transient (T). If the BBB or cAVB was still present on the follow-up ECG, it was considered persistent (P).

In order to study procedural related factors contributing to conduction disturbances, TAVI prostheses were divided into “balloon-expandable” (BE) and “self-expandable” (SE) prostheses, with BE prostheses being Edwards Sapien XT and -3 prostheses and SE prostheses comprising of Medtronic CoreValve, Medtronic Engager, Medtronic Evolut-R, St. Jude Medical Portico and Symetis Acurate.

### 2.3. Statistical Analysis

Categorical variables are presented as numbers, and proportions and binary logistic regression analysis were used to compare categorical variables with two categories and the χ^2^ test for categorical data with more than two categories.

For continuous variables, normality of distribution was assessed with the Kolmogorov–Smirnov test. Normal and skewed continuous variables are presented as means with standard deviation and medians with interquartile range, respectively. Differences between the groups were compared using the Kruskal–Wallis test, in case of a significant difference followed by a t-test while applying a Bonferroni correction for multiple comparisons. A two-side *p*-value < 0.05 was considered significant.

All statistical analyses were performed using Statistical Package for Social Sciences, version 23 (IBM SPSS, Chicago, IL, USA).

## 3. Results

**Figure 1:**
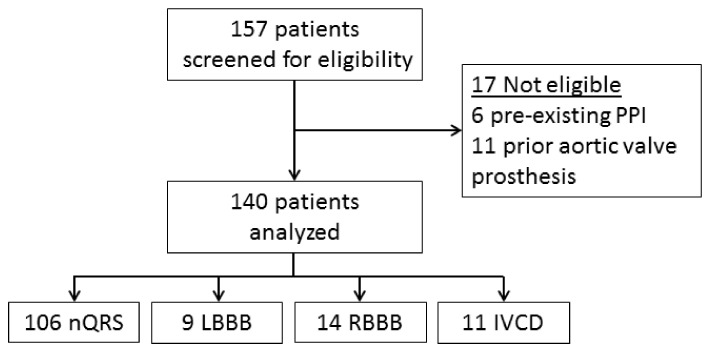
Study population. PPI: permanent pacemaker implantation; nQRS: narrow QRS; LBBB: left bundle branch block; RBBB: right bundle branch block; IVCD: intraventricular conduction disturbance.

A total of 157 patients were reviewed, of whom 17 were excluded because they did not fulfil the eligibility criteria. Therefore, 140 patients qualified for analysis, of whom 106 had a narrow QRS (nQRS) complex before the TAVI procedure (Figure 1). The baseline and procedural characteristics of the 140 patients are shown in Table 1.

### 3.1. Types of Conduction Disturbances

**Figure 2:**
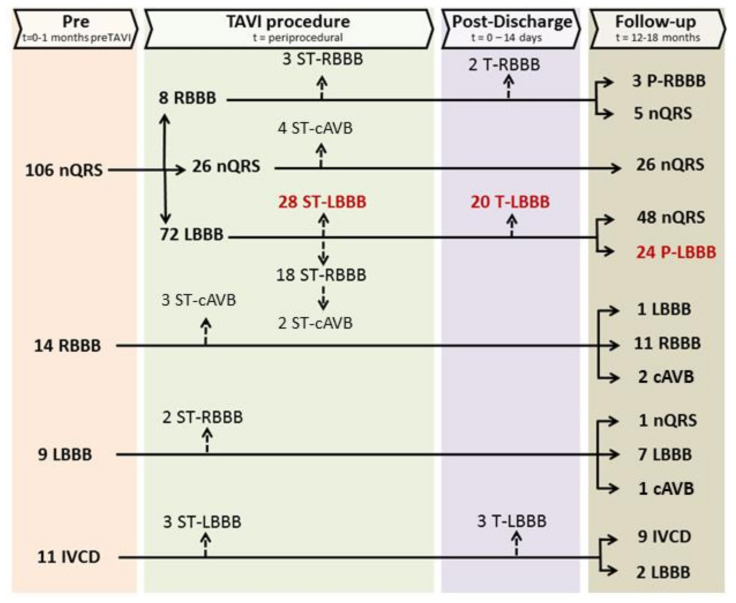
Types and dynamics of conduction disturbances in TAVI. Arrows within a ‘time-block’ show the dynamic nature of block development. Super-transient conduction disturbance is defined as any conduction disturbance present on the continuous electrocardiogram (ECG) during the transcatheter aortic valve implantation (TAVI) procedure which was not present on the first postoperative ECG. Transient conduction disturbance is defined as any new conduction disturbance present postoperative but disappeared at discharge or follow-up. Persistent conduction disturbance is defined as any new conduction disturbance present on the follow-up ECG. nQRS: narrow QRS; LBBB: left bundle branch block; RBBB: right bundle branch block; IVCD: intraventricular conduction disturbance; cAVB: third degree atrioventricular block; ST-: super transient-; T-: transient-; P-: persistent-.

**Figure 3:**
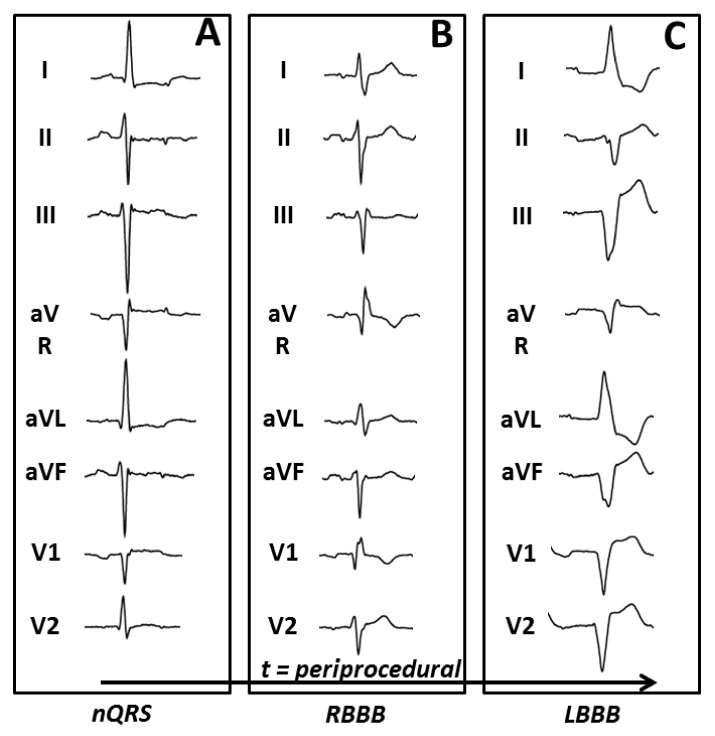
ECG example in a single study patient undergoing TAVI: ECG examples of a single patient with a baseline nQRS (**A**), converting periprocedural at insertion of the stiff wire into RBBB (**B**) and later switching to LBBB with PR prolongation after deployment of the TAVI (**C**). ECG: electrocardiogram; nQRS: narrow QRS complex; RBBB: right bundle branch block; LBBB: left bundle branch block.

Figure 2 shows the development of conduction disturbances throughout the TAVI procedure and follow-up. Among the 106 patients who started with a nQRS before TAVI (baseline), 72 (67.9%) patients developed a conduction disturbance; 28 (26.4%) patients developed a super-transient LBBB (ST-LBBB), 20 (18.9%) a transient LBBB (T-LBBB) and 24 (22.6%) a persistent LBBB (P-LBBB). Closer observation of the figure shows also that during the TAVI procedure many dynamic changes in conduction disturbances occurred, which were temporary in two-thirds of the cases. In eight nQRS patients a RBBB occurred, persisting in a third of them. Also, in 18 patients with a new LBBB, a temporary RBBB occurred (example shown in Figure 3, with two patients also having a temporary cAVB). Thirteen patients received a PPI at follow-up indicating progression of conduction disturbance. This was especially prominent in ST-LBBB patients (6/28) and P-LBBB patients (4/24).

Five out of the fourteen patients with a RBBB at baseline developed a cAVB, which was persistent in two patients and required a PPI. In the nine patients with a LBBB at baseline, two patients developed a ST-RBBB, one patient changed into nQRS and one patient developed a cAVB. In the 11 patients with an IVCD before TAVI, 8 developed a LBBB, being persistent in 2 patients. These alternating types of block show severe disturbance of the distal conduction pathways during the TAVI procedure.

### 3.2. Baseline and Procedural Characteristics

Table 2 shows baseline characteristics of the nQRS, ST-LBBB, T-LBBB and P-LBBB subgroups and indicates that a history of PCI and no-hypercholesterolemia significantly increased the chance of developing a P-LBBB. There were no differences in echocardiographic and CT-scan parameters between the subgroups.

With respect to procedural factors (Table 3), SE prostheses were nearly three times more likely to develop a P-LBBB than BE prostheses (*p* < 0.05). Implantation depth was approximately 2 mm more in P-LBBB patients than in the other three sub-groups, *p* = 0.12 (Table 3).

### 3.3. Time of Onset and Persistence of New LBBB and Cavb

**Figure 4:**
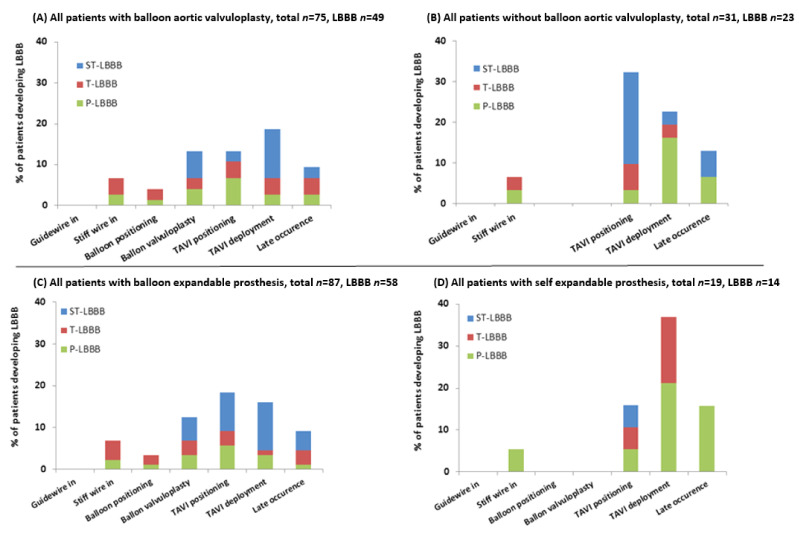
Distribution of first occurrence of new LBBB (in percentages) in patients with (**A**) and without BAV (**B**) and distribution of first occurrence of new LBBB (in percentages) in patients with balloon-expandable (**C**) and self-expandable valves (**D**). Distribution of first occurrence of new LBBB during the transcatheter aortic valve implantation (TAVI) procedure for patients who developed any LBBB for (**A**) all patients who had a balloon aortic valvuloplasty (BAV) prior to TAVI prosthesis implantation (*n* = 75) and (**B**) all patients who did not have such a BAV (*n* = 31). Distribution of first occurrence of new LBBB during the transcatheter aortic valve implantation (TAVI) procedure for patients who developed any LBBB for (**C**) all patients with a balloon-expandable prosthesis (*n* = 87) and (**D**) all patients with a self-expandable prosthesis (*n* = 19). LBBB: left bundle branch block; ST-: super transient-; T-: transient-; P-: persistent.

Figure 4A,B shows that no new LBBB was observed from baseline up to advancement of the guidewire through the aortic valve. In the patients where BAV was used (SE 8/19, BE 67/87), most LBBBs developed at BAV and at positioning and deployment of the TAVI prosthesis. The 31 patients who did not undergo BAV prior to TAVI implantation did not develop significantly less LBBBs during the entire procedure, (74.2% versus 65.3% for the noBAV and BAV groups, respectively; (*p* = 0.49) (Table 4)). This was due to a higher percentage of new LBBB acquired during positioning of the TAVI prosthesis in the noBAV compared to that in the BAV group (32.3% versus 13.3%, respectively (Figure 4A,B)). Furthermore, the noBAV and BAV groups did not significantly differ with respect to the development of ST-LBBB (32.3% vs. 24.0% (*p* = 0.47)), T-LBBB (12.9% vs. 21.3% (*p* = 0.42)) and P-LBBB (29.0% vs. 20.0% (*p* = 0.32)), respectively (Figure 4). Also, the persistency of LBBB was independent of the moment of development of the LBBB in patients with BAV; in patients without BAV most P-LBBB developed at TAVI deployment (Figure 4A,B).

Patients with a SE prosthesis have almost twice the risk of developing a new LBBB during TAVI deployment and at late occurrence compared to BE prostheses, with most being persistent (Figure 4C,D).

Approximately 15% of all new LBBBs occurred ‘late’, defined as after deployment of the TAVI prosthesis but before the end of the procedure. The patients with a SE prosthesis seemed to have almost double the risk of developing such a late conduction disturbance (4/19) compared to BE prostheses (11/87) (Figure 4C,D).

All cAVBs occurred at positioning or deployment of the BAV or TAVI prosthesis. Four PPI occurred before discharge and nine at follow-up.

## 4. Discussion

The present study shows the high frequency and dynamic nature of conduction disturbances developing during TAVI. In the entire cohort only 32% of patients did not develop any conduction block during the TAVI procedure, while 23% developed a persistent left bundle branch block and 3% a persistent right bundle branch block. The remaining 45% developed various forms of transient blocks. The study shows the dynamicity of development of conduction disturbances by the frequent switching between LBBB, RBBB, cAVB and nQRS during the procedure. Importantly, particular P-LBBB and ST-LBBB patients progressed into AVB, requiring PPI. Furthermore, the percentage of LBBB is similar in patients with and without BAV, suggesting that the development of BBB is (almost) inevitable in a subpopulation of patients following TAVI procedure. This may be explained by the fact that we described that the effective distance between the aortic valve and conduction system (EDACS) predicts LBBB, which is a patient-specific anatomy [15].

### 4.1. The Dynamics of TAVI-Induced Conduction Blocks

The continuous ECG monitoring in the present study has elucidated the dynamics of conduction disturbances during the TAVI procedure, as evidenced by any conduction abnormality in ~70% of patients and even alternating blocks within the same patient. Besides the previously reported T-LBBB and P-LBBB [16], we now demonstrate that a quarter of all patients develop a ST-LBBB during the TAVI procedure.

Striking was also the occurrence of new RBBB, with or without alternation to LBBB or cAVB. The occurrence of RBBB has been previously reported but with very low incidence [17]. The higher incidence in our study can be explained by also including ST-RBBBs. Because the right bundle branch itself is not close to the aortic valve, direct contact of the prosthesis and the right bundle branch is unlikely. However, it is known that within the His bundle the fibers of the right (RBB) and left bundle branch (LBB) are already separated [18,19]. Therefore, TAVI-induced RBBB may be related to the damage caused by the prosthesis to the area of the His bundle containing RBB fibers. The alternation between RBBB, LBBB and cAVB may be explained by minute motion of prosthesis parts near the proximal rapid conduction system. Considering that the thickness of the bundle of His is only a few millimeters, small changes following contact with the prosthesis can affect one or both branches (inducing LBBB or RBBB) with potential for cAVB.

Almost a quarter of all patients developed a P-LBBB with SE prostheses being particularly prone to persistency. Follow-up of one year showed that 16.7% (4/24) of patients with P-LBBB required a PPI at follow-up. Interestingly, an even larger percentage of patients with a ST-LBBB (21.4% (6/28)) required a PPI, showing that this subgroup should receive more intensive follow-up.

### 4.2. Potential Mechanisms of Conduction Disturbances Induced by TAVI

There is little evidence concerning the exact mechanism for the development of conduction blocks following TAVI. Apparently, no animal studies are available to formulate suitable hypotheses. Our observations may help shed some lights on potential causes for these events.

Most conduction blocks developed during the actual TAVI with resolution in the majority of cases by the end of the procedure. Only a minority of blocks developed after the procedure or even days later.

The rapid development of the blocks may be caused by direct physical damage or compression-induced ischemic damage to the cells of the conduction system or interstitial edema. Permanent mechanical or ischemic damage cannot recover within hours, because myocytes, which are responsible for impulse propagation, do not divide. On the other hand, temporary ischemia may be caused by initial myocardial compression and subsequent settling of the prosthesis in the aortic root. Alternatively, damage may have caused swelling of the extracellular space, which results in disruption of the gap junctions [20,21,22] and may resolve within minutes to hours. The few slower developing conduction blocks may be elicited by gradually increasing ischemia and/or inflammatory and degenerative processes.

### 4.3. Luxating Factors of New LBBB and Predictors for Persistency

The observation that the total number of TAVI-induced LBBBs in BAV and noBAV patients was similar, suggests that there are patients who are susceptible to any manipulation at the aortic annulus, regardless of whether this is from BAV or TAVI positioning. This suggests an anatomical substrate for TAVI-induced conduction blocks. Such substrate may be related to specific anatomy of the membranous septum [23], pre-existent conduction delays as demonstrated in electrophysiology studies with an increased HV-interval [24,25] or the presence of calcifications and other pathological disturbances [11]. Better recognition of such a substrate could reduce the number of TAVI-induced conduction disturbances and thereby the outcome of the procedure.

Besides the presence of a patient-specific substrate, Table 3 shows that almost half of all SE prostheses produced a P-LBBB, indicating also prosthesis-specific luxating factors. Deeper implantation of the prosthesis into the left ventricular outflow tract can explain why these SE prostheses are at risk of persistent conduction disturbances [3,6,26,27]. Other investigators have also proposed that the self-expanding frame of such prostheses could induce, besides the reported late conduction blocks, persistent conduction blocks [16,28,29].

The observation that most LBBBs and cAVBs were luxated at BAV and at positioning and deployment of the TAVI prosthesis corresponds with the findings of Nuis et al. [8]. In addition, the proportion of temporary versus permanent LBBB appears similar in our BAV group to that in their study. These investigators studied 65 patients who received BAV prior to SE prosthesis implantation. The difference between this study and ours is that most of our patients received BE prostheses. The difference in prosthesis types used may also explain why the percentage of new LBBBs (excluding ST-LBBB) after BAV in our study are only a quarter of those reported by Nuis et al. [8] (~10% compared to 40%), as the BE prostheses create 2–3 times less conduction disturbances [30].

### 4.4. Study Limitations

Because of the observational design, our study could be hampered by information and selection bias. Still, all data were prospectively collected from a consecutive series of patients and entered in a central database with established definitions.

As patients undergoing TAVI are increasingly younger and fitter, not only the procedure itself with associated conduction disturbances are important to assess but also preventive (traditional) cardiovascular risk factors such as smoking on TAVI outcomes will become more important and thus advisable for future research [31,32].

### 4.5. Clinical Implications

Continuous measurement of the ECG during TAVI is useful because important conduction abnormalities such as ST-LBBB can be detected. ST-LBBB may be a warning sign for progression to cAVB at follow-up, thus warranting adequate rhythm monitoring postoperative and in the outpatient care setting. An implantable loop-recorder could be a good interim solution until further data are available for selecting the correct patients who require a pacemaker to prevent them from cAVB-caused bradycardia.

## 5. Conclusions

This study shows the high frequency of conduction blocks developing during TAVI and their dynamic nature, with most blocks disappearing within hours. The alternating pattern between LBBB, RBBB and progression to cAVB suggests involvement of the His bundle in the pathophysiology.

Important novel observations in this study were that the use of BAV does not influence the occurrence of LBBB, but that the use of SE prostheses does. Moreover, ST-LBBB is not innocent, because it has a high risk of developing cAVB, therefore, perhaps indicating the need for PPI.

## Figures and Tables

**Table 1:** Baseline characteristics all patients and procedural factors.

	*n* = 140
Age (years)	81 [78–84]
Female (*n*)	75 (53.6)
BMI (kg/m^2^)	26.3 [23.9–29.3]
Logistic EuroScore (%)	10.0 [7.6–15.8]
EuroScore II (%)	2.7 [1.6–4.1]
Additive Score (%)	8.0 [7.0–10.0]
AF (*n*)	55 (39.3)
NYHA (*n*)	
I	3 (2.1)
II	40 (28.6)
III	92 (65.7)
IV	5 (3.6)
AMI (*n*)	24 (17.1)
PCI (*n*)	47 (33.6)
CABG (*n*)	24 (17.1)
CVA (*n*)	13 (9.3)
TIA (*n*)	15 (10.7)
PAD (*n*)	27 (19.3)
Carotid disease (*n*)	25 (17.9)
Diabetes (*n*)	52 (37.1)
Creatinin (µmol/L)	91 [77–114]
COPD (*n*)	21 (15.0)
Hypertension (*n*)	135 (96.4)
Hypercholesterolemia (*n*)	130 (92.9)
	
ECG	
PR interval pre (ms)	164 [133–190]
QRS duration pre (ms)	97 [88–120]
	
Echocardiogram	
LVEF (%)	58 [50–64]
Mean Gradient (mmHg)	45.0 ± 14.3
Peak Gradient (mmHg)	74.8 ± 21.4
Aortic Valve Area (cm^2^)	0.8 [0.6–0.9]
	
CT scan	
Aortic annulus-short axis (cm)	2.2 [2.1–2.4]
Aortic annulus-Long axis (cm)	2.8 [2.6–2.9]
Aortic annulus–Circumference (cm)	8.0 ± 0.9
Aortic annulus–Area (cm^2^)	4.8 ± 0.9
Aortic annulus-Effective diameter (cm)	2.5 [2.3–2.6]
Aortic annulus-Perimeter derived diameter (cm)	2.5 [2.4–2.7]
	
Procedural factors	
Access Route	
Trans-femoral (*n*)	105 (75.0)
Trans-apical (*n*)	33 (23.6)
Trans-aortic (*n*)	2 (1.4)
	
Prosthesis type	
Balloon-expandable (*n*)	117 (83.6)
Self-expandable (*n*)	23 (16.4)
	
Prosthesis size	
20 mm (*n*)	3 (2.1)
23 mm (*n*)	35 (25.0)
25 mm (*n*)	3 (2.1)
26 mm (*n*)	66 (47.1)
27 mm (*n*)	1 (0.7)
29 mm (*n*)	32 (22.9)
	
Balloon aortic valvuloplasty (*n*)	99 (70.7)
	
Prosthesis depth	
At Septum (mm)	6.3 [5.1–8.6]
At Lateral wall (mm)	5.7 [4.2–7.3]
	
PPI	18 (12.9)

Results are presented as median (interquartile range), mean ± standard deviation or absolute number (percentage). BMI: body mass index; AF: atrial fibrillation; NYHA: New York Heart Association; AMI: acute myocardial infarction; PCI: percutaneous coronary intervention; CABG: coronary artery bypass grafting; CVA: cerebrovascular accident; TIA: transient ischemic attack; PAD: peripheral arterial disease; COPD: chronic obstructive pulmonary disease; ECG: electrocardiogram; PRpre interval: PR interval preoperative; QRSpre duration: QRS duration preoperative; LVEF: left ventricular ejection fraction; CT: computerized tomography; PPI: permanent pacemaker implantation.

**Table 2:** Baseline characteristics of LBBB groups.

Baseline Characteristics	nQRS (*n* = 34)	ST-LBBB (*n* = 28)	T-LBBB (*n* = 20)	P-LBBB (*n* = 24)	*p* Value
Age (years)	80.00 [74.75–85.00]	80.00 [76.50–84.00]	81.50 [78.00–83.00]	83.00 [77.75–85.00]	0.549
Female (*n*)	21 (61.8)	11 (39.3)	13 (65.0)	17 (70.8)	0.100
BMI (kg/m^2^)	26.17 [23.26–29.32]	26.47 [23.08–30.96]	26.38 [24.10–31.70]	26.12 [24.05–27.66]	0.795
Logistic EuroScore (%)	10.90 [7.69–16.66]	9.02 [6.89–11.74]	9.33 [6.86–11.24]	11.93 [8.28–19.39]	0.187
EuroScore II (%)	3.01 [1.95–3.95]	2.52 [1.56–3.36]	2.07 [1.55–3.45]	2.82 [1.62–5.76]	0.133
Additive Score (%)	9.00 [7.00–10.00]	8.00 [7.00–8.75]	8.00 [7.00–8.00]	8.50 [8.00–10.00]	0.187
AF (n)	16 (47.1)	11 (39.3)	5 (25.0)	9 (37.5)	0.457
NYHA (*n*)					0.050
I	0 (0.0)	0 (0.0)	0 (0.0)	3 (12.5)	
II	9 (26.5)	6 (21.4)	8 (40.0)	5 (20.8)	
III	24 (70.6)	22 (78.6)	10 (50.0)	15 (62.5)	
IV	1 (2.9)	0 (0.0)	2 (10.0)	1 (41.7)	
AMI (*n*)	5 (14.7)	5 (17.9)	1 (5.0)	2 (8.3)	0.507
PCI (*n*)	9 (26.5)	11 (39.3)	2 (10.0)	13 (54.2)	0.013
CABG (*n*)	7 (20.6)	2 (7.1)	1 (5.0)	5 (20.8)	0.208
CVA (*n*)	5 (14.7)	3 (10.7)	1 (5.0)	2 (8.3)	0.701
TIA (*n*)	5 (14.7)	3 (10.7)	1 (5.0)	3 (12.5)	0.746
PAD (*n*)	9 (26.5)	7 (25.0)	3 (15.0)	4 (16.7)	0.677
Carotid disease (*n*)	8 (23.5)	5 (17.9)	0 (0.0)	6 (25.0)	0.116
Diabetes (*n*)	12 (35.3)	10 (35.7)	9 (45.0)	11 (45.8)	0.783
Creatinin (µmol/L)	88.50 [77.75–115.50]	91.50 [73.50–109.50]	91.50 [77.25–109.00]	91.50 [77.25–114.25]	0.298
COPD (*n*)	6 (17.6)	6 (17.6)	4 (20.0)	4 (16.7)	0.970
Hypertension (*n*)	33 (97.1)	28 (100.0)	19 (95.0)	23 (95.8)	0.725
Hypercholesterolemia (*n*)	34 (100.0)	27 (96.4)	19 (95.0)	19 (79.2)	0.013
					
ECG					
PR interval pre (ms)	96.00 [88.00–105.00]	91.00 [80.50–100.00]	89.00 [86.00–94.00]	90.00 [86.00–100.00]	0.202
QRS duration pre (ms)	169.00 [153.0–191.0]	177.00 [159.00–190.50]	175.00 [159.00–190.00]	172.00 [143.00–195.00]	0.675
					
Echocardiogram					
LVEF (%)	58.00 [49.00–65.00]	59.50 [50.75–63.00]	59.50 [55.00–65.00]	58.50 [57.00–66.75]	0.814
MG (mmHg)	40.00 (15.48)	51.00 (17.56)	45.00 (10.96)	44.00 (14.00)	0.874
PG (mmHg)	74.50 (22.86)	77.00 (24.71)	75.00 (16.10)	70.50 (21.79)	0.870
AVA (cm^2^)	0.80 [0.60–1.00]	0.80 [0.60–0.90]	0.70 [0.55–1.00]	0.70 [0.50–0.89]	0.538
					
CT scan					
AN-short axis (cm)	2.20 [2.08–2.40]	2.20 [2.04–2.30]	2.25 [2.03–2.40]	2.10 [2.00–2.55]	0.727
AN-Long axis (cm)	2.70 [2.50–2.90]	2.80 [2.60–3.02]	2.70 [2.60–2.89]	2.75 [2.50–2.90]	0.381
AN–Circumference (cm)	7.80 (0.87)	8.00 (0.75)	7.85 (1.31)	7.90 (0.69)	0.494
AN–Area (cm^2^)	4.70 (1.06)	4.71 (0.95)	4.60 (0.74)	4.70 (0.79)	0.883
AN-Effective diameter (cm)	2.50 [2.30–2.53]	2.45 [2.25–2.60]	2.43 [2.28–2.63]	2.50 [2.30–2.63]	0.951
AN-Perimeter derived diameter (cm)	2.48 [2.30–2.64]	2.50 [2.40–2.73]	2.60 [2.50–2.75]	2.55 [2.33–2.68]	0.499

Results are presented as median (interquartile range), mean ± standard deviation or absolute number (percentage). BMI: body mass index; AF: atrial fibrillation; NYHA: New York Heart Association; AMI: acute myocardial infarction; PCI: percutaneous coronary intervention; CABG: coronary artery bypass grafting; CVA: cerebrovascular accident; TIA: transient ischemic attack; PAD: peripheral arterial disease; COPD: chronic obstructive pulmonary disease; ECG: electrocardiogram; PRpre interval: PR interval preoperative; QRSpre duration: QRS duration preoperative; LVEF: left ventricular ejection fraction; MG: mean gradient; PG: peak gradient; AVA: aortic valve area; CT: computerized tomography; AN: aortic annulus.

**Table 3:** Procedural factors for all LBBB groups.

Procedural Factors	nQRS (*n* = 34)	ST-LBBB (*n* = 28)	T-LBBB (*n* = 20)	P-LBBB (*n* = 24)	*p* Value
Access Route					0.124
TF (*n* = 79)	20 (25.3)	22 (27.8)	17 (21.5)	20 (25.3)	
TA (*n* = 26)	14 (53.8)	5 (19.2)	3 (11.5)	4 (15.4)	
TAO (*n* = 1)	0 (0.0)	1 (3.6)	0 (0.0)	0 (0.0)	
					
Prosthesis type *					0.014
Balloon-expandable (*n* = 87)	29 (33.3)	27 (31.0)	16 (18.4)	15 (17.2)	
Self-expandable (*n* = 19)	5 (26.3)	1 (5.3)	4 (21.1)	9 (47.4)	
					
Prosthesis size *					0.181
20	1 (100.0)	0 (0.0)	0 (0.0)	0 (0.0)	
23	13 (41.9)	7 (22.6)	3 (9.7)	8 (25.8)	
25	0 (0.0)	1 (100.0)	0 (0.0)	0 (0.0)	
26	15 (30.6)	15 (30.6)	11 (22.4)	8 (16.3)	
27	0 (0.0)	0 (0.0)	0 (0.0)	1 (100.0)	
29	5 (21.7)	5 (21.7)	6 (26.1)	7 (30.4)	
					
Balloon aortic valvuloplasty (*n* = 75)	26 (34.7)	18 (24.0)	16 (21.3)	15 (20.0)	0.446
					
Prosthesis depth					
At Septum (mm)	6.14 [5.09–8.13]	6.18 [4.63–8.32]	6.32 [4.83–7.18]	8.34 [5.67–10.72]	0.122
At Lateral wall (mm)	5.62 [4.05–7.09]	5.79 [4.33–6.85]	4.83 [4.40–7.61]	6.42 [3.79–9.61]	0.556
					
PPI (*n* = 13)	1 (7.7)	6 (46.2)	2 (15.4)	4 (30.8)	0.144

* Percentages were calculated per row. Results are presented as absolute number (percentage) or (in case of prosthesis depth) as median (interquartile range). TF: transfemoral; TA: trans-apical; TAO: trans-aortic; PPI: permanent pacemaker implantation.

**Table 4:** Risk of development of LBBB in procedures with and without balloon aortic valvuloplasty.

	BAV (*n* = 75)	noBAV (*n* = 31)	*p*-Value
nQRS (*n*)	26 (34.7)	8 (25.8)	0.49
ST-LBBB (*n*)	18 (24.0)	10 (32.3)	0.47
T-LBBB (*n*)	16 (21.3)	4 (12.9)	0.42
P-LBBB (*n*)	15 (20.0)	9 (29.0)	0.32

Results are presented as absolute number (percentage). LBBB: left bundle branch block; ST-: super transient-; T-: transient-; P-: persistent; nQRS: narrow QRS complex; BAV: balloon aortic valvuloplasty; noBAV: no balloon aortic valvuloplasty.

## Data Availability

Not applicable.
